# Supplementary material for: Land use change and El Niño-Southern Oscillation drive decadal carbon balance shifts in Southeast Asia
Source: Nat Commun. 2018 Mar 20;9:1154. doi: 10.1038/s41467-018-03374-x (PMC5861034; doi:10.1038/s41467-018-03374-x)
Supplement: Supplementary file 1 — Supplementary Information(PDF 2557 kb) [file 41467_2018_3374_MOESM1_ESM.pdf]

Supplementary Information to "Land use change and El Niño-Southern Oscillation drive decadal carbon balance shifts in Southeast Asia"

Masayuki Kondo[1,2], Kazuhito Ichii[1,2,3], Prabir K. Patra[2], Joseph G. Canadell[4], Benjamin Poulter[5,6], Stephen Sitch[7], Leonardo Calle[5], Yi Y. Liu[8,9], Albert I. J. M. van Dijk[10], Tazu Saeki[3], Nobuko Saigusa[3], Pierre Friedlingstein[7], Almut Arneth[11], Anna Harper[7], Atul K. Jain[12], Etsushi Kato[13], Charles Koven[14], Fang Li[15], Thomas A. M. Pugh[11,16], Sönke Zaehle[17], Andy Wiltshire[18], Frederic Chevallier[19], Takashi Maki[20], Takashi Nakamura[21], Yosuke Niwa[20], Christian Rödenbeck[17]

[1]Center for Environmental Remote Sensing (CEReS), Chiba University, Chiba 263-8522, Japan

[2]Department of Environmental Geochemical Cycle Research, Japan Agency for Marine-Earth Science and Technology, Yokohama 236-0001, Japan

[3]Center for Global Environmental Research, National Institute for Environmental Studies, Tsukuba 305-8506, Japan

[4]Global Carbon Project, CSIRO Oceans and Atmosphere, Canberra, Australian Capital Territory 2601, Australia

[5]Institute on Ecosystems and Department of Ecology, Montana State University, Bozeman, Montana 59717, USA

[6]NASA Goddard Space Flight Center, Biospheric Science Laboratory, Greenbelt, Maryland 20771, USA

[7]University of Exeter, Exeter EX4 4QF, UK

[8]School of Geography and Remote Sensing, Nanjing University of Information Science and Technology, Nanjing, 210044, China

[9]ARC Centre of Excellence for Climate Systems Science & Climate Change Research Centre, University of New South Wales, Sydney, New South Wales 2052, Australia

[10]Fenner School of Environment & Society, Australian National University, Canberra, Australian Capital Territory 0200, Australia

[11]Institute of Meteorology and Climate Research, Environmental Atmospheric Research (IMK-IFU), Karlsruhe Institute of Technology (KIT), Kreuzeckbahnstraße 19, 82467 Garmisch-Partenkirchen, Germany

[12]Department of Atmospheric Sciences, University of Illinois at Urbana-Champaign, Urbana, Illinois 61801, USA

[13]Institute of Applied Energy, Tokyo, 105-0003, Japan

[14]Earth Sciences Division, Lawrence Berkeley National Laboratory, Berkeley, CA 94720, USA

[15]International Center for Climate and Environmental Sciences, Institute of Atmospheric Physics, Chinese Academy of Sciences, Beijing 100864, China

[16]School of Geography, Earth & Environmental Science and Birmingham Institute of Forest Research, University of Birmingham, B15 2TT, UK

[17]Biogeochemical Integration Department, Max Planck Institute for Biogeochemistry, 07701 Jena, Germany

[18]Met Office Hadley Centre, Fitzroy Road, Exeter EX1 3PB, UK

[19]Laboratoire des Sciences du Climat et de l’Environnement (LSCE), CEA CNRS UVSQ, 91191 Gif Sur Yvette, France

[20]Meteorological Research Institute, Tsukuba 305-0052, Japan

[21]Japan Meteorological Agency, Tokyo 100-8122, Japan

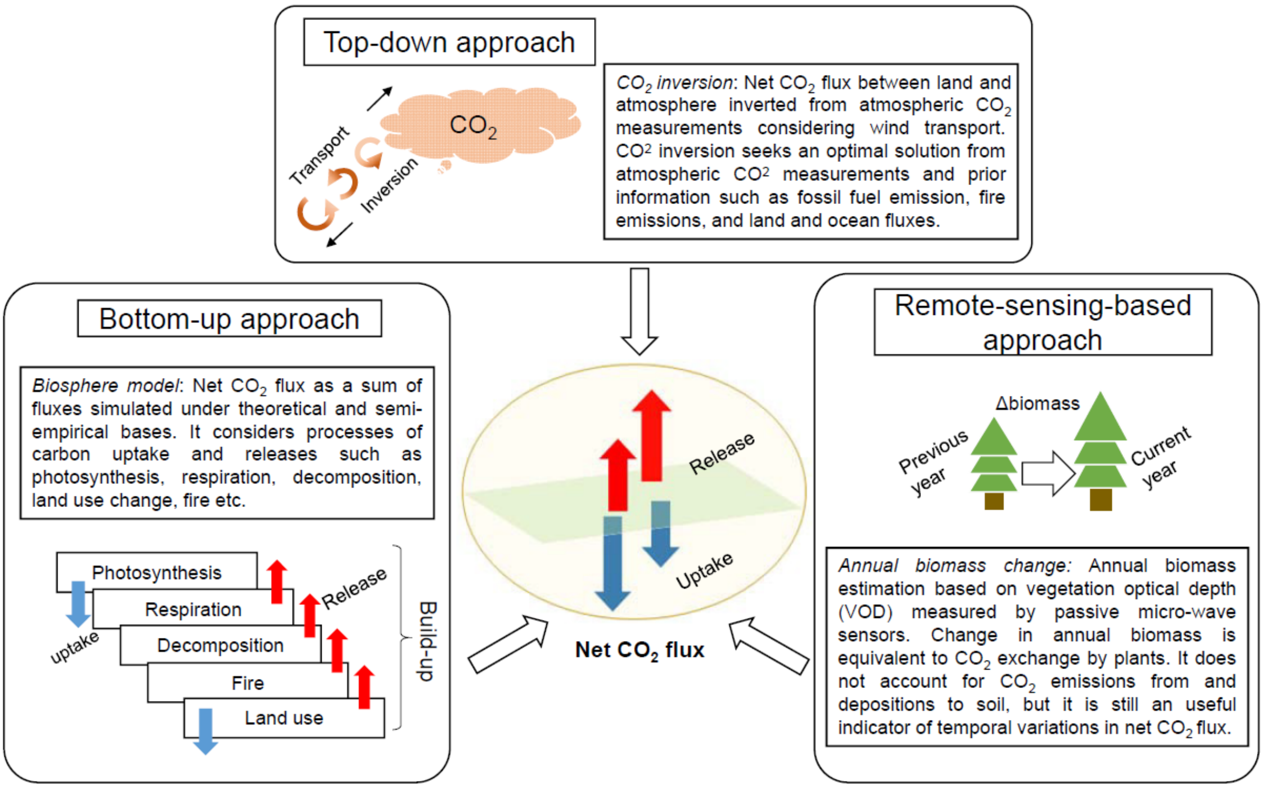

Supplementary Figure 1. Conceptual figure for net CO_2 flux estimations analysed in this study: bottom-up (process-based biosphere model), top-down (atmospheric CO_2 inversion), and remote-sensing-based (Δbiomass) approaches.

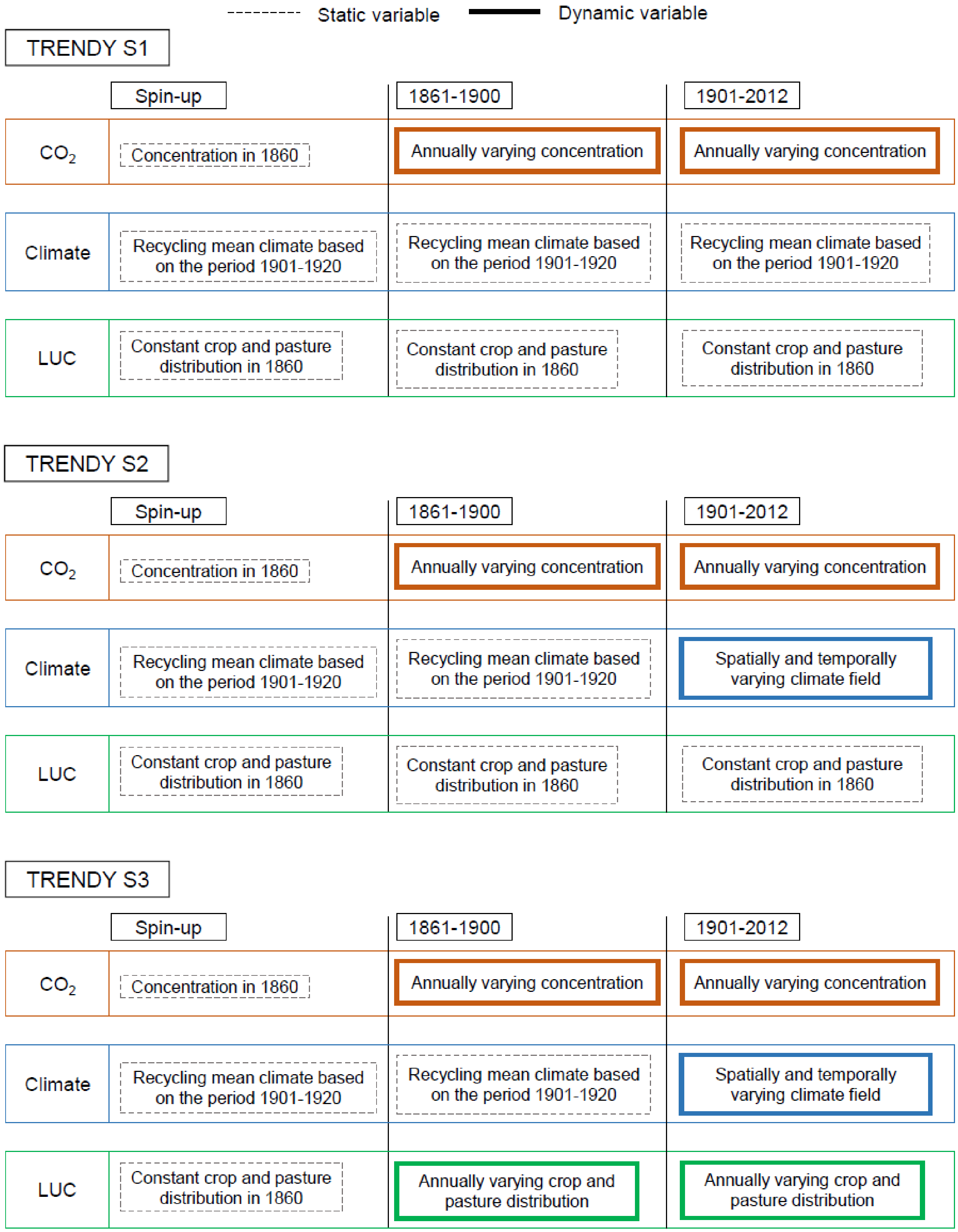

Supplementary Figure 2. Configuration of the forcing data (CO_2, climate, and LUC) for the TRENDY simulations (TRENDY S1, S2, and S3).

a

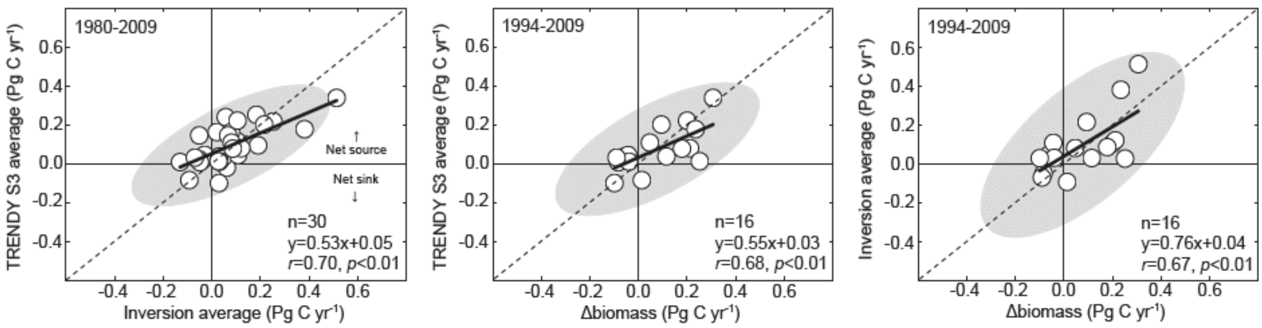

b

	CLM	ISAM	JULES	LPJ	LPJ-GUESS	O-CN	VISIT	Average
CLM	1							
ISAM	0.59**	1						
JULES	0.36*	0.63**	1					
LPJ	0.40*	0.58**	0.54**	1				
LPJ-GUESS	0.48**	0.61**	0.69**	0.79**	1			
OCN	0.39*	0.70**	0.46**	0.78**	0.58**	1		
VISIT	0.68**	0.85**	0.74**	0.73**	0.69**	0.75**	1	
Average	0.77**	0.83**	0.73**	0.81**	0.83**	0.76**	0.94**	1

n=30 for all entries

c

	ACTM	JENA	JMA	MACC	NICAM	Average
ACTM	1					
JENA	0.46* (n=20)	1				
JMA	0.54* (n=20)	0.27 (n=25)	1			
MACC	0.56** (n=20)	0.37* (n=25)	0.24 (n=25)	1		
NICAM	0.48* (n=18)	0.57** (n=20)	0.42* (n=20)	0.34 (n=20)	1	
Average	0.64** (n=20)	0.57** (n=30)	0.61** (n=25)	0.73** (n=30)	0.90** (n=20)	1

Supplementary Figure 3. a, Scatter plots between an ensemble average of annual NBP by the TRENDY S3, that by the atmospheric CO_2 inversions, and Δbiomass. All relationships are shown along with the 95% confident ellipses and regression lines. These scatter plots correspond to the inset of Figure 1a. Correlation matrices for **b,** annual NBP of seven biosphere models from the TRENDY S3 (for the period 1980−2009) and **c,** of five atmospheric CO_2 inversions (for overlapping simulation periods; see Supplementary Table 2 for simulation periods). Statistical significances are indicated by ** ($p < 0.01$) and * ($p < 0.05$).

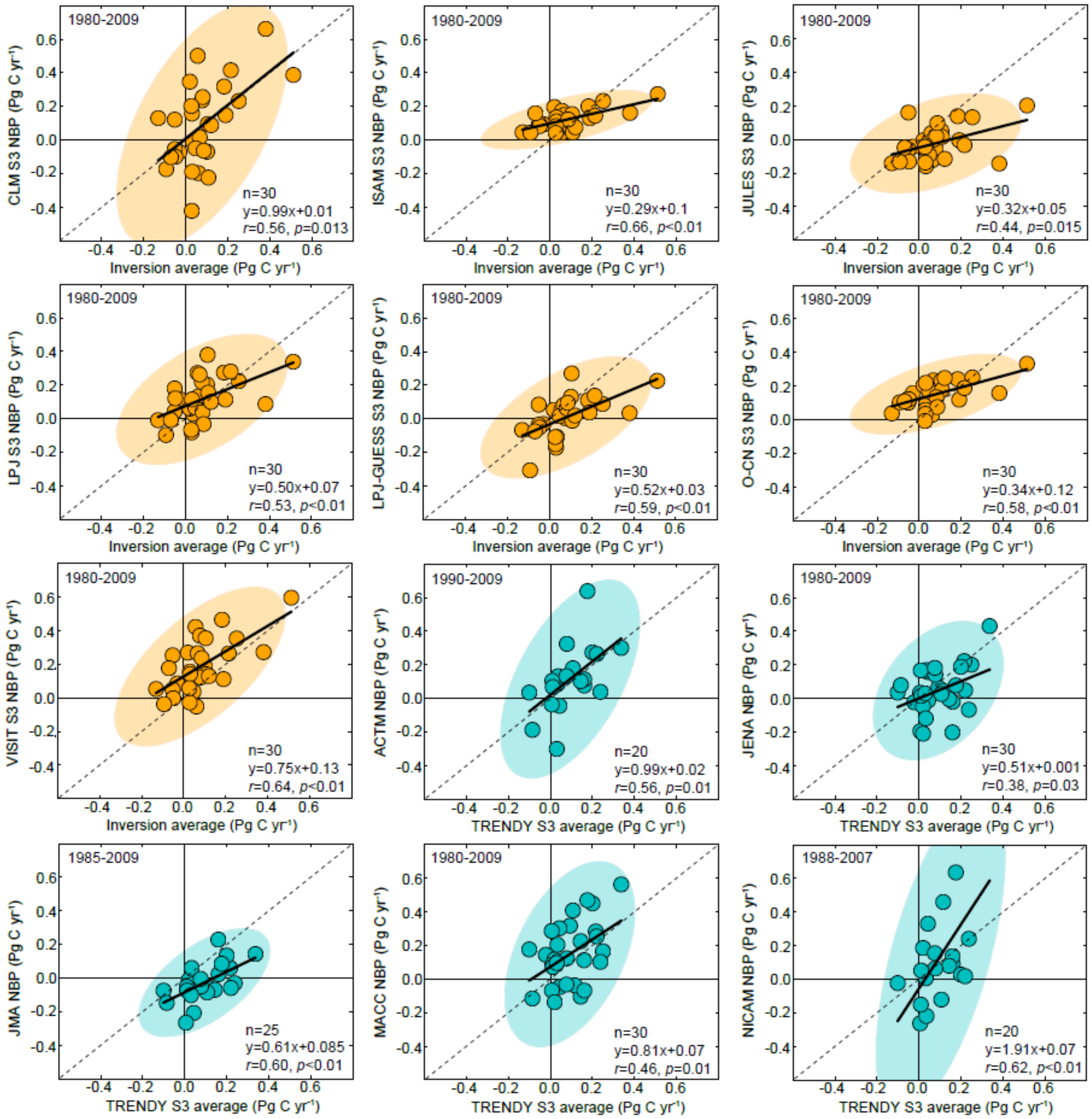

Supplementary Figure 4. Scatter plots of annual NBP between seven biosphere models from the TRENDY S3 and an ensemble average of the atmospheric CO_2 inversions (orange circles), and between five atmospheric CO_2 inversions and an ensemble average of biosphere models from the TRENDY S3 (cyan circles). All relationships are shown along with the 95% confident ellipses and regression lines.

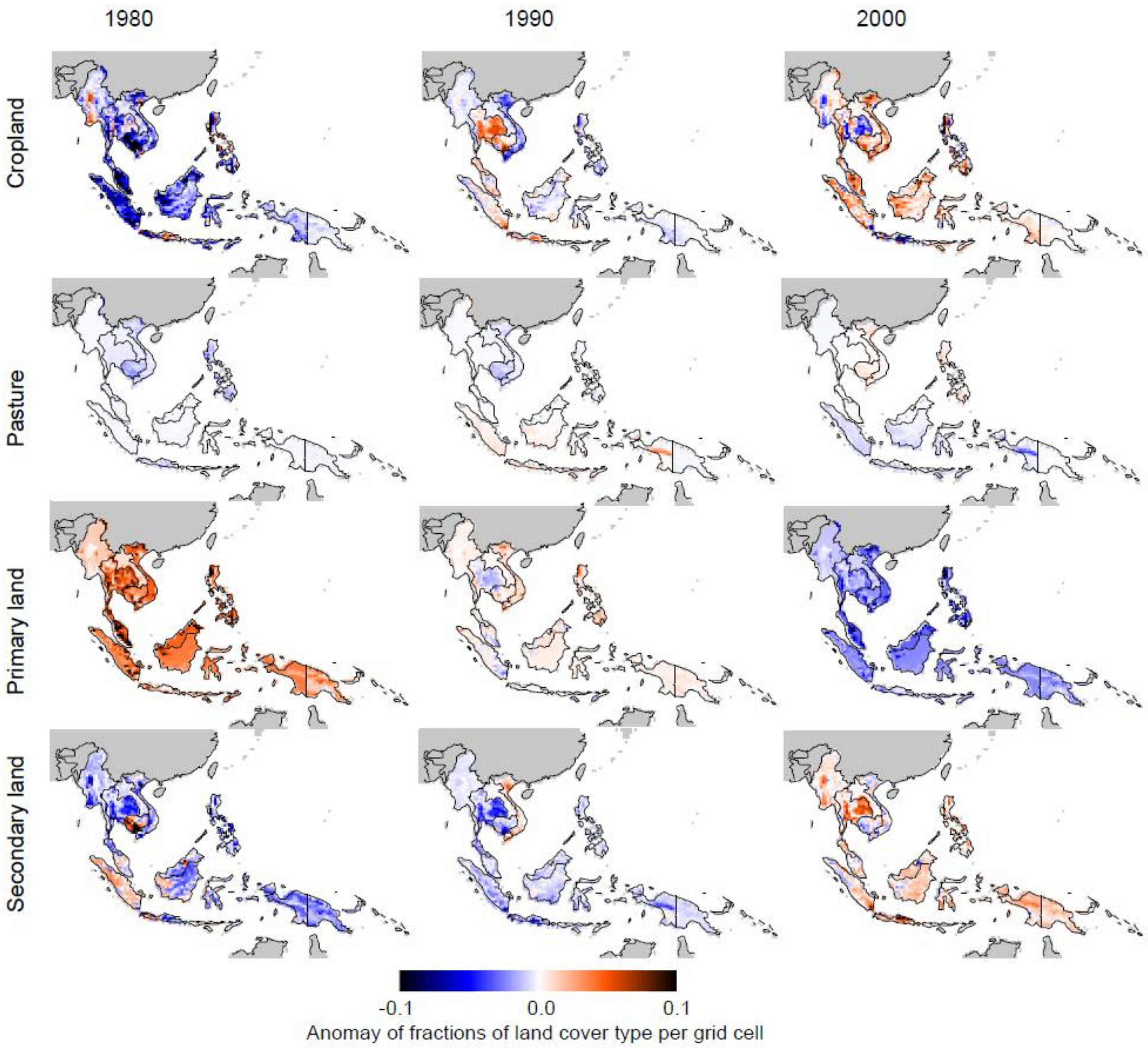

Supplementary Figure 5. Spatial patterns in fractions of land cover type (i.e, cropland, pasture, primary and secondary lands) of the LUC forcing data in the year 1980, 1990, and 2000. These patterns are shown in anomalies with a base period 1980−2009.

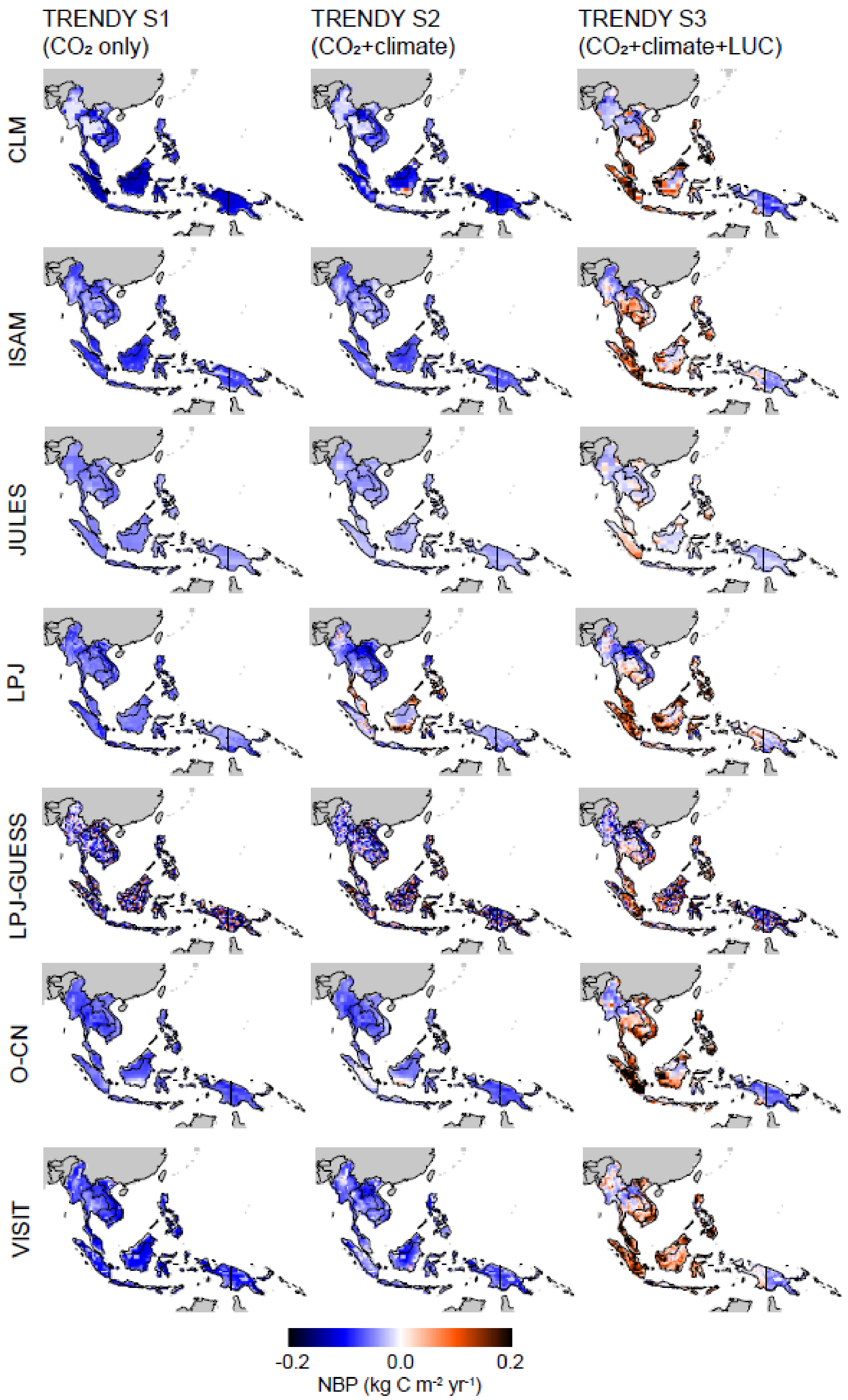

Supplementary Figure 6. Spatial distributions of mean annual NBP from the TRENDY S1, S2, and S3 for seven TRENDY models for the period 1980−2009. It should be noted that heterogeneous spatial patterns of LPJ-GUESS are due to the model's unique characteristic, so-called forest gap dynamics, which involves a number of stochastic processes related to plant mortality.

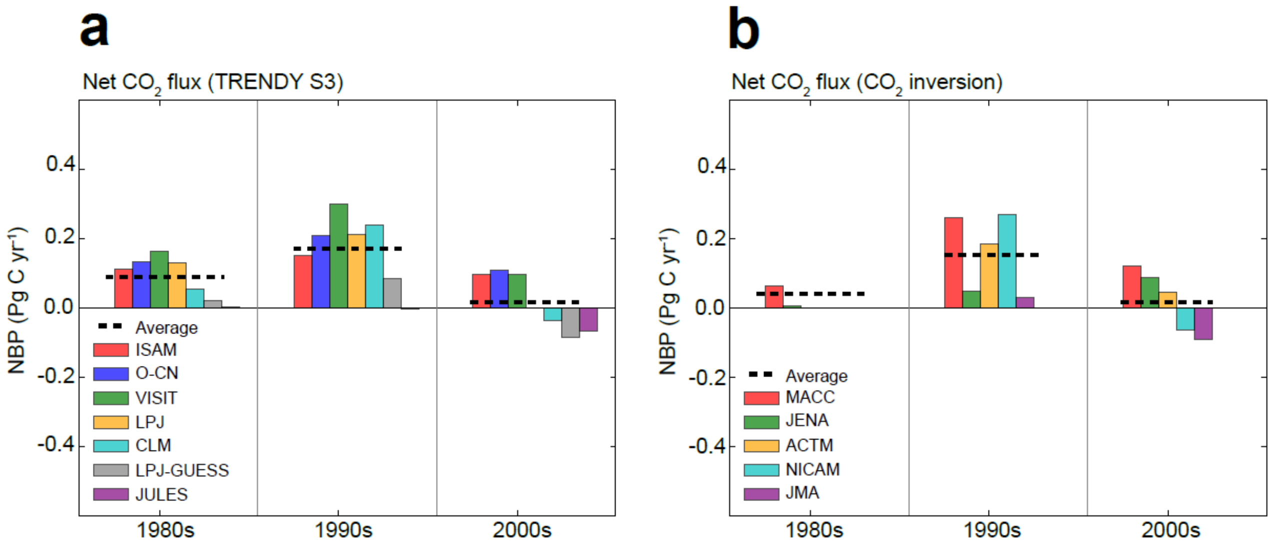

Supplementary Figure 7. Inter-decadal variability in NBP by individual models from **a,** the TRENDY S3 and **b,** atmospheric CO_2 inversions, along with ensemble averages of individual estimates (dashed horizontal lines).

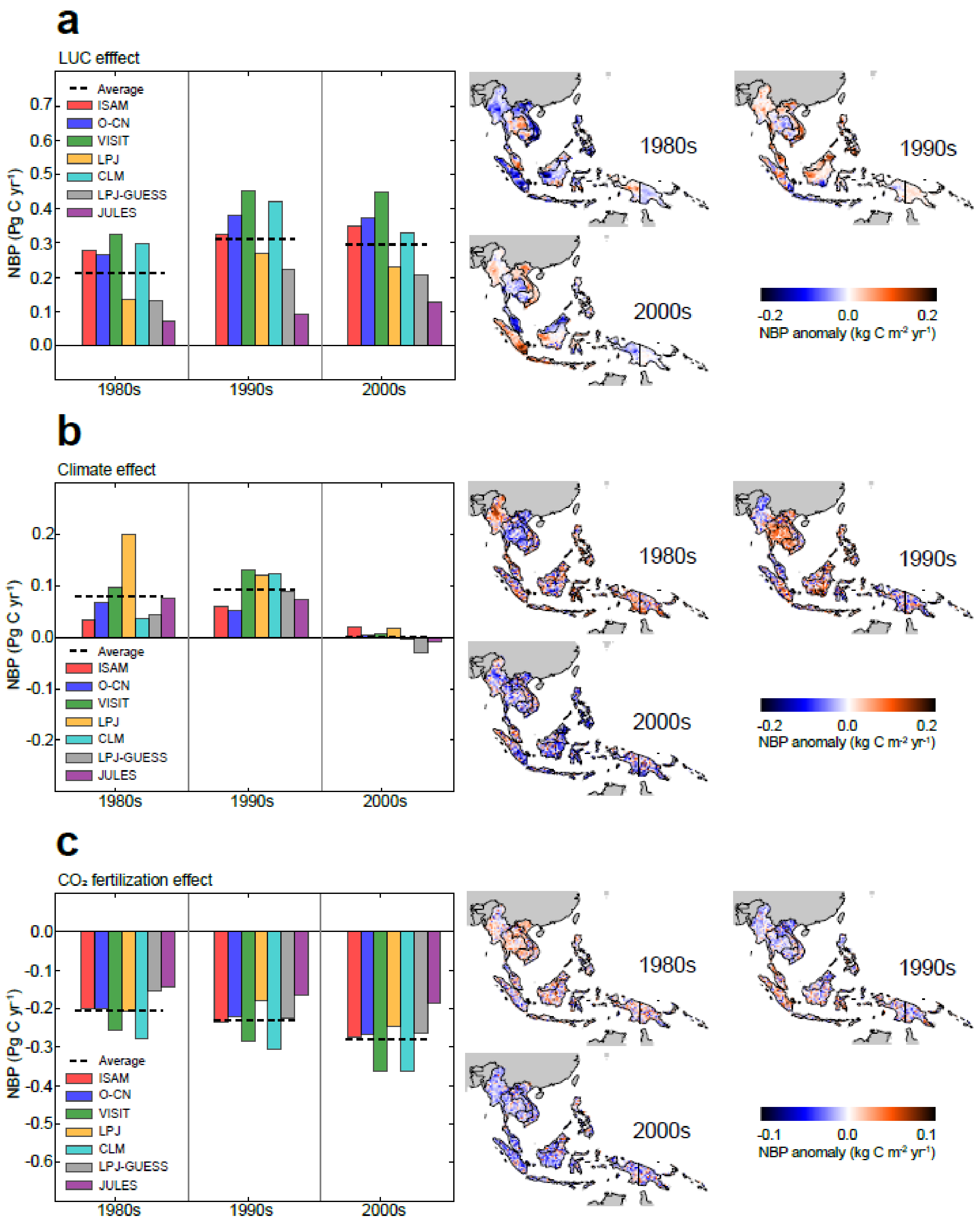

Supplementary Figure 8. Individual TRENDY model results for attributions to NBP: **a,** the LUC, **b,** climate, and **c,** CO_2 fertilization effects, along with ensemble averages of individual estimates (dashed horizontal lines), and spatial variability of decadal NBP (the 1980s, 1900s, and 2000s) for each attribution. The spatial variability shown are anomalies from the ensemble average of the TRENDY model simulations with a base period 1980−2009.

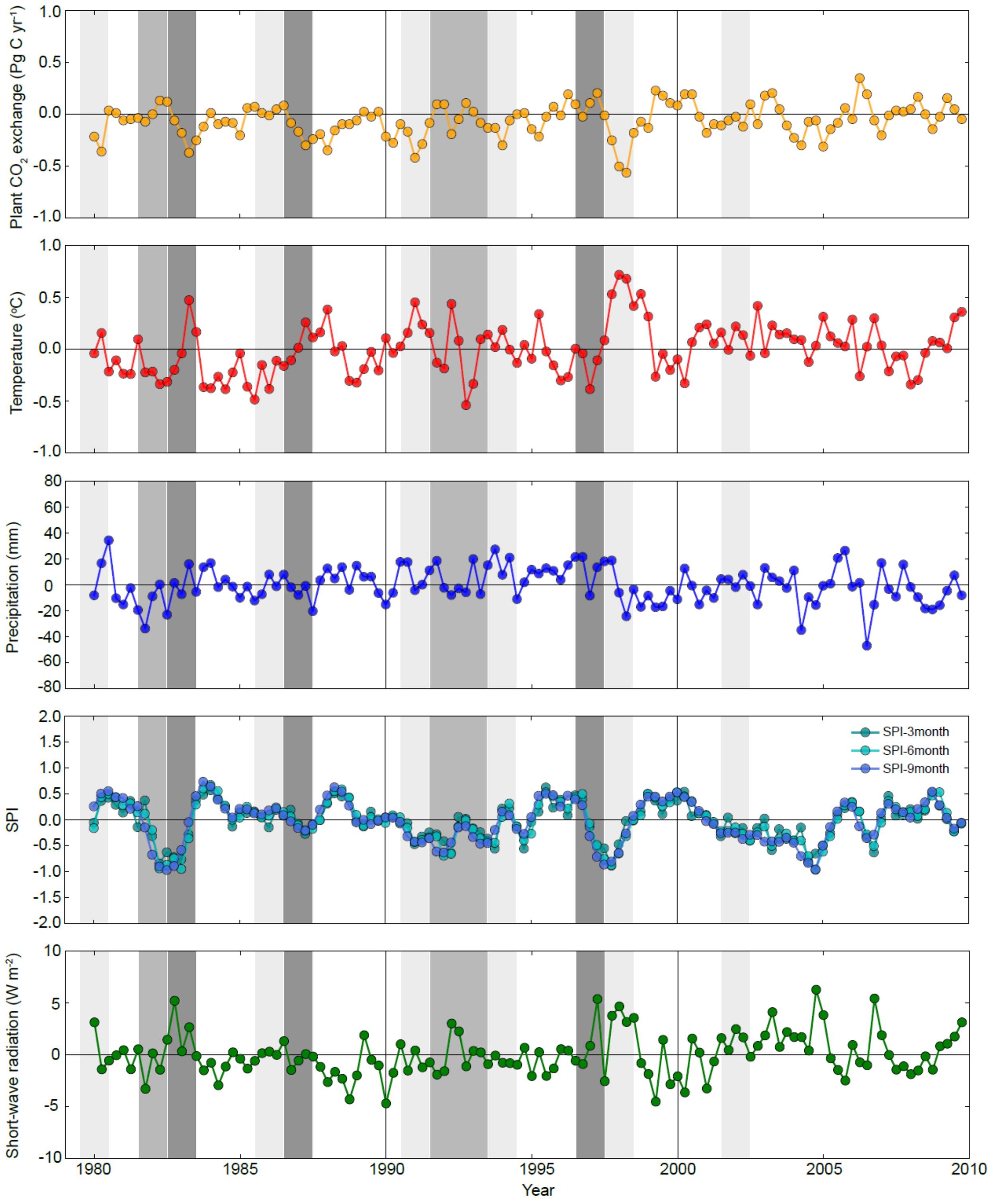

Supplementary Figure 9. Seasonal anomalies (three month averages) of plant CO_2 exchange by the ensemble of TRENDY models, temperature, precipitation, SPIs (3-, 6-, and 9-month), and short-wave radiation for the period 1980–2009 (a base period 1980–2009). Grey shading represents higher MEI values.

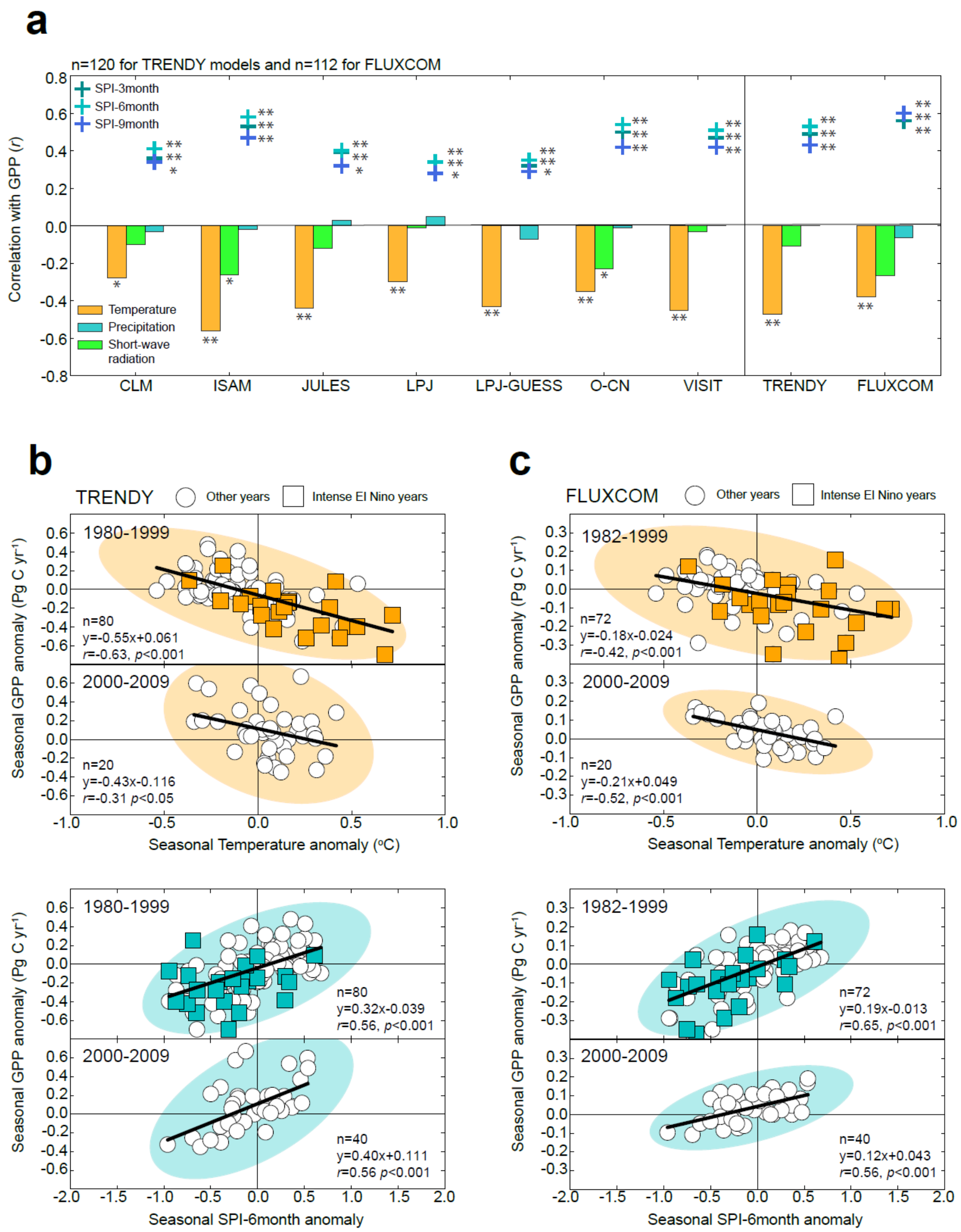

Supplementary Figure 10. a, Correlation coefficients (*r*) in relationship between seasonal anomalies of GPP induced by the climate effect (TRENDY S2-S1) and climate variables (temperature, precipitation, short-wave radiation, and three types of SPIs) for the period 1980−2009. Statistical significances are indicated by ** (p < 0.01) and * (p < 0.05). Relationships between seasonal anomalies of GPP and temperature, and SPI-6 month for the periods 1980−1999 and 2000−2009, for **b,** the TRENDY and **c**, FLUXCOM. The figure configuration is the same as in Fig. 5.

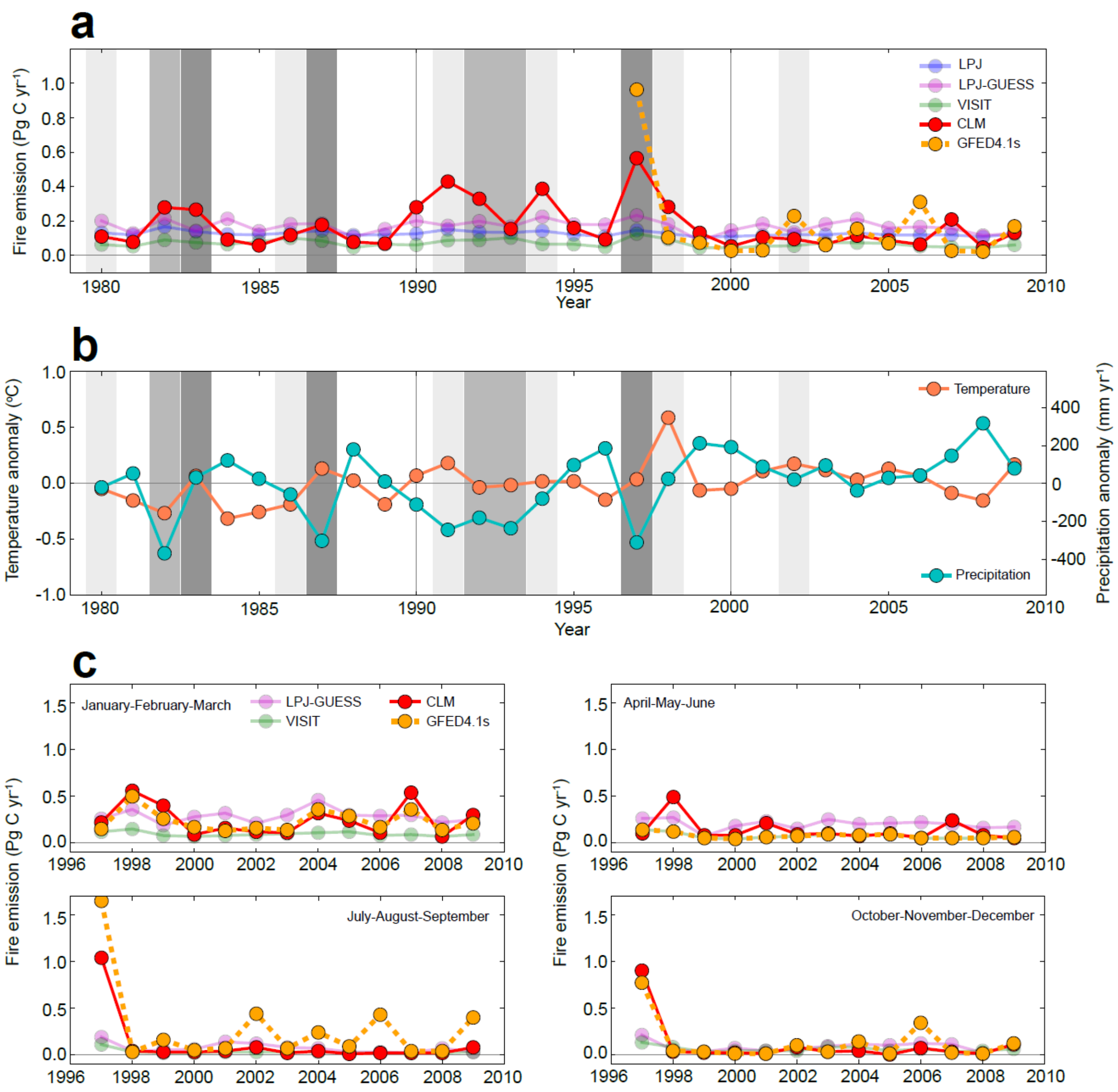

Supplementary Figure 11. a, Comparison between interannual variability in fire emissions from the TRENDY models and GFED4.1s (orange dashed line). A result of the CLM model (the only model considers peat and deforestation fires among the TRENDY models) is indicated by a red line, and other models by opaque coloured lines. **b,** Interannual variability in temperature and precipitation anomalies from CRU-NCEP dataset (a base period 1980−2009). Colour of grey shading represents the strength of MEI values. **c,** Seasonal fire emissions from the estimates in **a** (except LPJ, monthly fire emissions are not available in the TRENDY-v2 dataset).

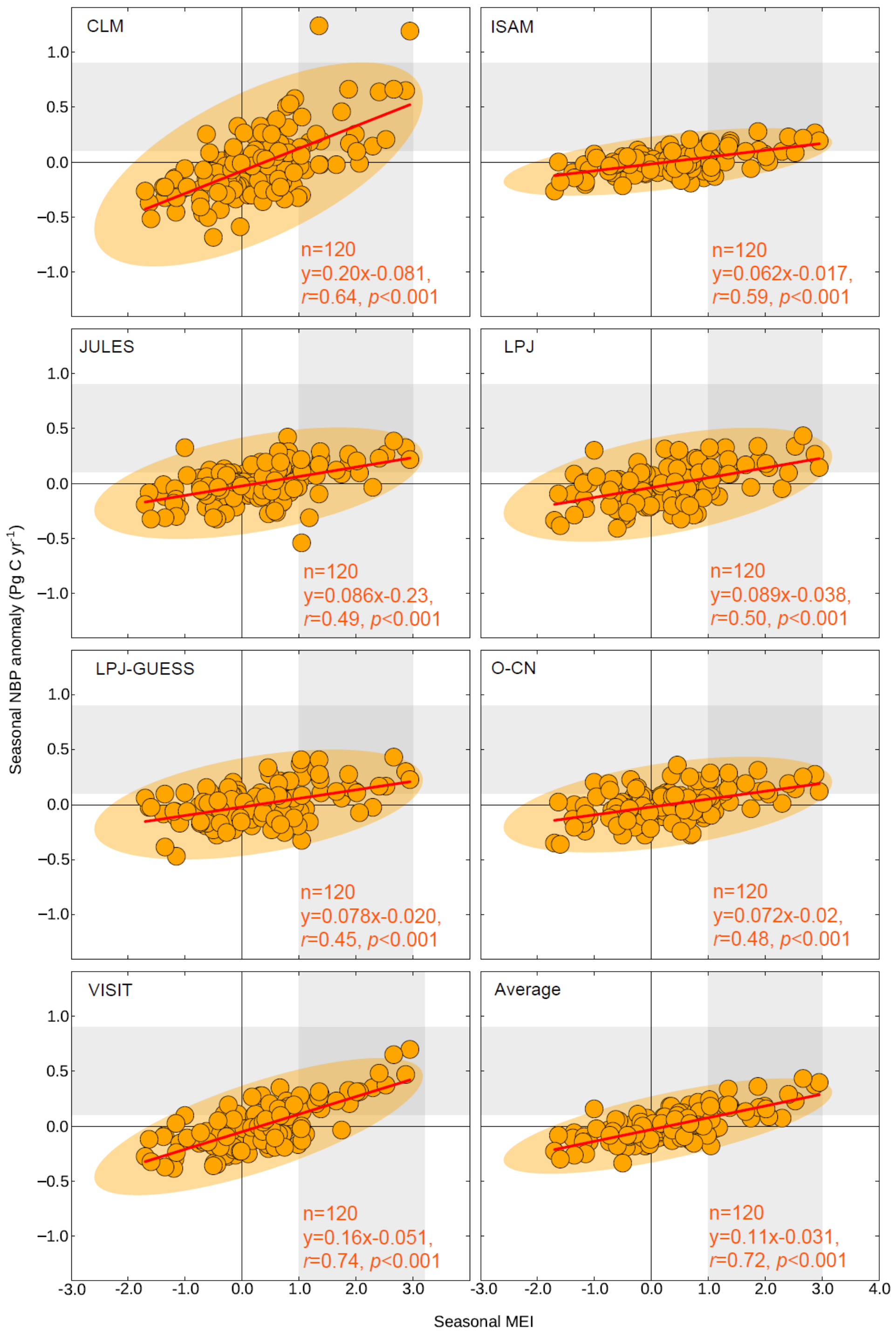

Supplementary Figure 12. Relationships between seasonal MEI and seasonal NBP anomaly from the TRENDY S3 for the period 1980−2009. These relationships are constructed in such a way that MEI leads NBP anomaly by there months. The results are shown for seven individual models and their ensemble average. All relationships are shown along with the 95% confident ellipses and regression lines.

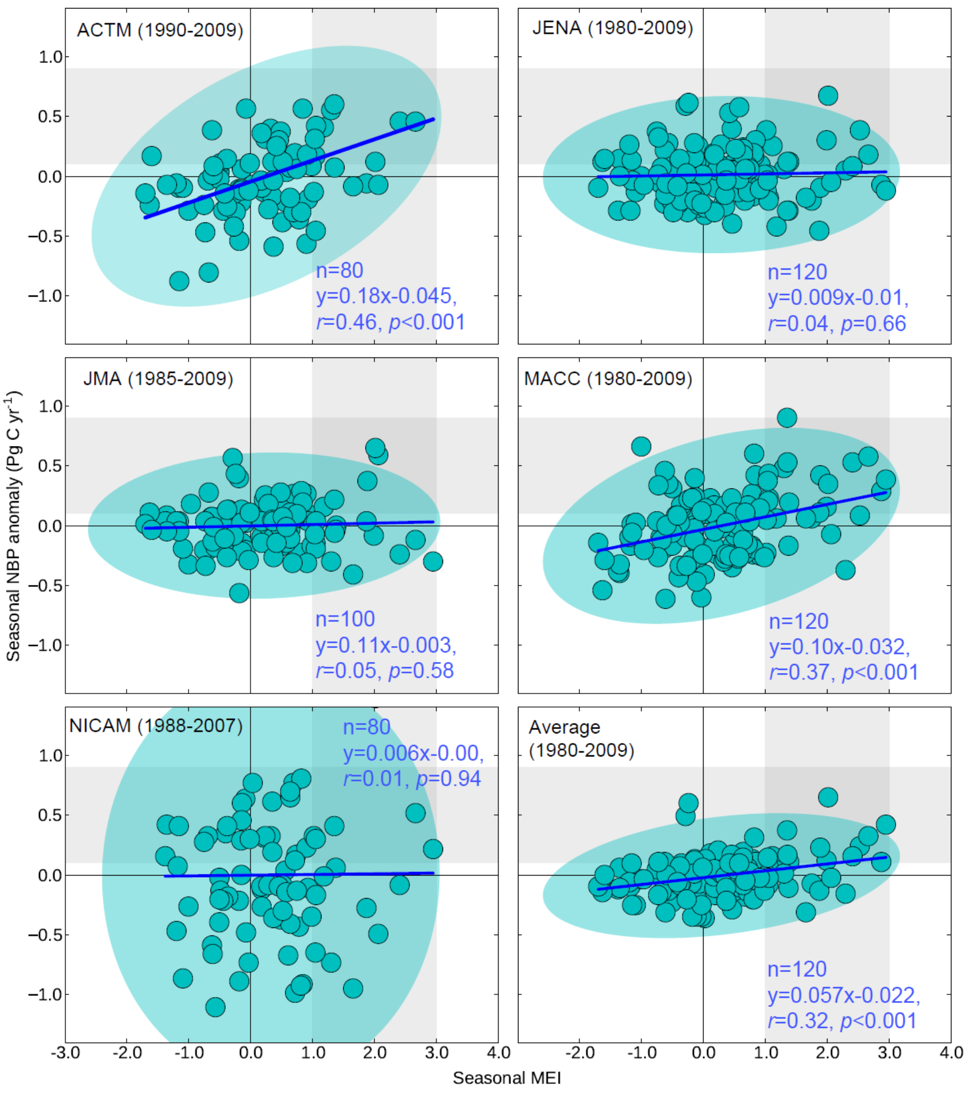

Supplementary Figure 13. Relationships between seasonal MEI and seasonal NBP anomaly from the atmospheric CO_2 inversions. These relationships are constructed in such a way that MEI leads NBP anomaly by there months. The results are shown for five individual models (the period differs by models) and their ensemble average (for the period 1980–2009). All relationships are shown along with the 95% confident ellipses and regression lines.

Supplementary Table 1. Configuration of the TRENDY models used in the analyses.

Biosphere model (Abbreviation in the text)	Spatial resolution	Dynamic vegetation	Carbon-Nitrogen coupling	Fire simulation (including peat fire)	Land use change scheme		
					Distinction between primary and secondary lands	Wood harvest	Crop harvest
CLM4.5 (CLM)	2.5˚ × 2.5˚	Yes	Yes	Yes (Yes)	Yes	Yes	Yes
ISAM	0.5˚ × 0.5˚	Yes	Yes	No	Yes	Yes	Yes
JULES ver.3.2 (JULES)	5˚ × 3.75˚	Yes	No	No	No	No	No
LPJwsl (LPJ)	0.5˚ × 0.5˚	Yes	No	Yes (No)	No	No	Yes*
LPJ-GUESS	0.5˚ × 0.5˚	Yes	No	Yes (No)	Yes	No	Yes*
O-CN	1˚ × 1˚	No	Yes	No	No	Yes	Yes
VISIT	0.5˚ × 0.5˚	No	No	Yes (No)	Yes	Yes	Yes

*Simulated as managed grasslands, with grass harvest when Leaf Area Index (LAI) reached its maximum.

Supplementary Table 2. Configuration of the atmospheric CO_2 inversion systems used in the analyses

Inversion system (Abbreviation in the text)	Number of regions	Time period	IAV wind*	IAV prior#	Number of observations	Reference
ACTM v5.7b (ACTM)	84	1990–2011	Yes	Yes: FF, LA<br>No: BB, SA	73 (GLOBALVIEW)	(59)
JENA s81-v3.8 (JENA)	Grid-cells	1980–2014	Yes	Yes: FF<br>No: BB, LA, SA	15 (from various institutions)	Update of (60)
JMA_CDTM (JMA)	22	1985–2012	Yes	Yes: FF<br>No: BB, LA, SA	90 (WDCGG)<br>16 (aircraft observations),<br>58 (vessel observations)	(61)
MACC v14r2 (MACC)	Grid cells (3.75° × 2.5°)	1979–2014	Yes	Yes: FF<br>No: BB, LA, SA	81 (NOAA ESRL)	(62)
NICAM-TM (NICAM)	40	1988–2007	Yes	Yes: FF<br>No: BB, LA, SA	59 (GLOBALVIEW),<br>12 (CONTRAIL)	(63)

*Inversion accounts for interannual variability of wind (Yes/No). #Inversion accounts for interannual variability of prior fluxes (Yes/No). Abbreviations: fossil-fuel emission (FF), biomass burning emission (BB), land-air CO_2 exchange (LA), and sea-air CO_2 exchange (SA).

Supplementary Table 3. Configuration of transport model, meteorology data for transport, prior fluxes used in atmospheric CO_2 inversion systems.

Inversion system	Transport model	Meteorology	Prior flux			
			Land	Ocean	Biomass Burning	Fossil fuel emissions
ACTM	Atmospheric Chemistry-Transport Model (ACTM)	NCEP2	Three-hourly flux from CASA	Month flux from the LDEO (Takahashi) Surface pCO_2 database	--	EDGAR v4.2 rescaled global total to CDIAC
JENA	Tracer Transport Model version 3 (TM3)	ERA	Time-mean spatial pattern from LPJ	A combination of multiple sources	--	EDGAR v4.2 rescaled global total to the BP statistical Review of World Energy
JMA	JMA atmospheric transport model	JRA-25 (up to 2006), and JCDAS thereafter	Three-hourly flux from CASA	Month flux from the LDEO (Takahashi) Surface pCO_2 database	--	TransCom3 fixed distribution, scaled global total to CDIAC
MACC	Tracer Transport Model version 5 (TM5)	ECMWF	Three-hourly flux from ORCHIDEE	Month flux from the LDEO (Takahashi) Surface pCO_2 database	GFAS	EDGAR v4.2 rescaled global total to CDIAC
NICAM	Nonhydorstatic ICosahedral Atmospheric Model (NICAM)	JCDAS	Three-hourly flux from CASA	Month flux from the LDEO (Takahashi) Surface pCO_2 database	GFED3	EDGAR v4.1 rescaled global total to CDIAC

Supplementary Table 4. Optimal relationships between seasonal MEI and seasonal NBP anomaly for each model from the TRENDY S3 and atmospheric CO_2 inversions. Relationships that yielded the highest correlation (r) in the range of three- to nine-month lags between MEI and NBP (MEI forwards NBP) are shown for each model. Statistical significances are indicated by ** ($p < 0.001$) and * ($p < 0.01$).

	Lag (months)	Linear regression	Correlation (r)
***TRENDY S3** (period)*			
CLM (1980−2009)	Three	y = 0.20x - 0.081	0.64** (n=120)
ISAM (1980−2009)	Three	y = 0.062x - 0.017	0.59** (n=120)
JULES (1980−2009)	Three	y = 0.089x - 0.23	0.49** (n=120)
LPJ (1980−2009)	Six	y = 0.093x - 0.032	0.53** (n=120)
LPJ-GUESS (1980−2009)	Three	y = 0.078x - 0.02	0.45** (n=120)
O-CN (1980−2009)	Three	y = 0.072x + 0.02	0.48** (n=120)
VISIT (1980−2009)	Three	y = 0.11x + 0.039	0.74** (n=120)
***Atmospheric CO_2 inversion** (period)*			
ACTM (1990−2009)	Three	y = 0.18x - 0.045	0.46** (n=80)
JENA (1980−2009)	Nine	y = 0.058x - 0.018	0.25* (n=120)
JMA (1985−2009)	Nine	y = 0.072x - 0.011	0.32** (n=100)
MACC (1980−2009)	Three	y = 0.10x - 0.032	0.37** (n=120)
NICAM (1988−2007)	Nine	y = 0.20x - 0.077	0.33* (n=80)
